# Supplementary figures and images for: Childhood immune imprinting to influenza A shapes birth year-specific risk during seasonal H1N1 and H3N2 epidemics
Source: PLoS Pathog. 2019 Dec 19;15(12):e1008109. doi: 10.1371/journal.ppat.1008109 (PMC6922319; doi:10.1371/journal.ppat.1008109)

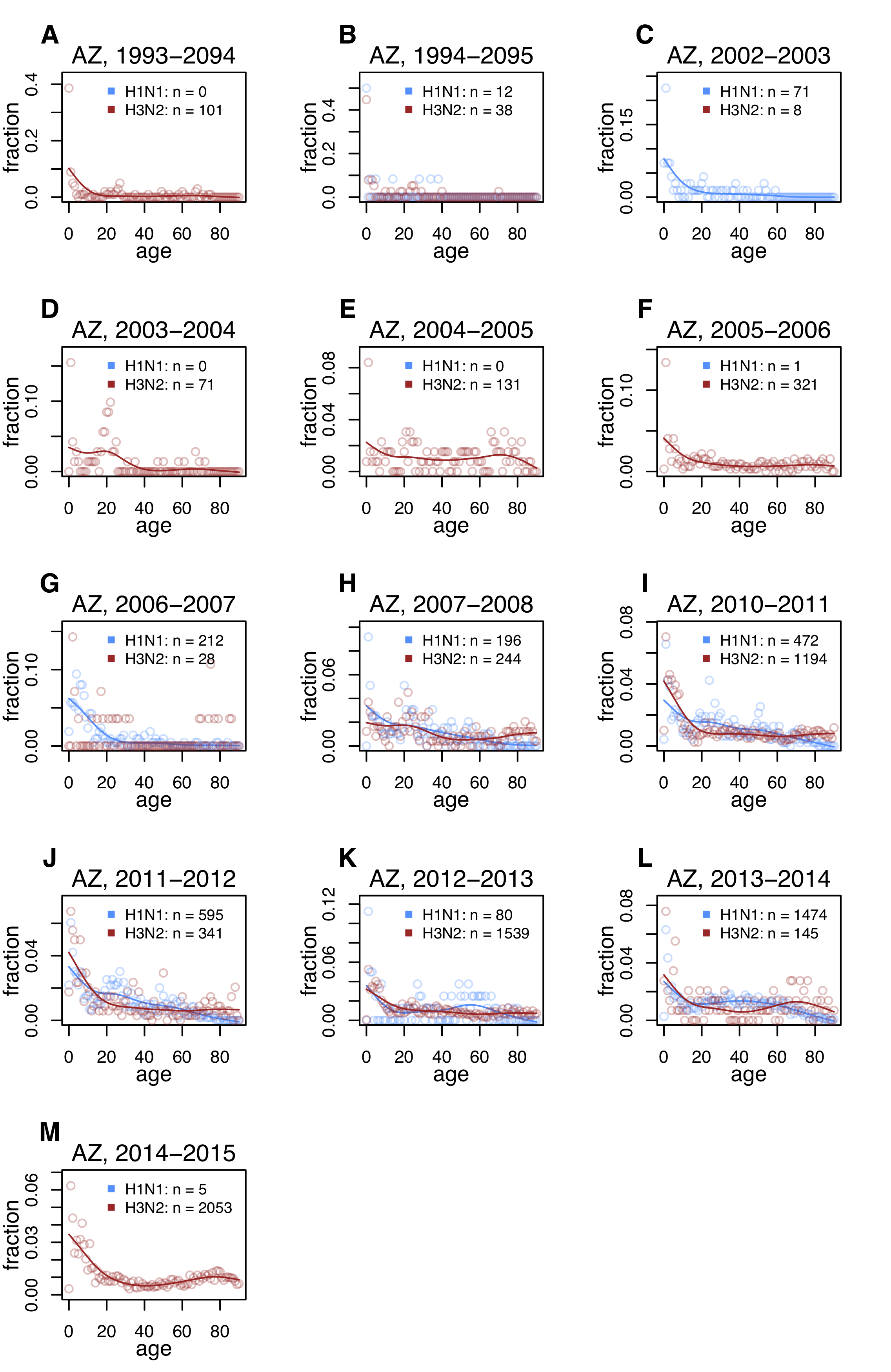

Supplement: S1 Fig — Supplement to Fig 2 showing observed age distributions from all influenza seasons. Observed case fractions (points) were only plotted if 10 or more cases of a given subtype were confirmed, to avoid extreme stretching of the y axis. Smoothing splines were only plotted if 50 or more cases of a given subtype were observed, as fits to fewer data points would not have been meaningful. (TIFF) [file ppat.1008109.s001.tiff]

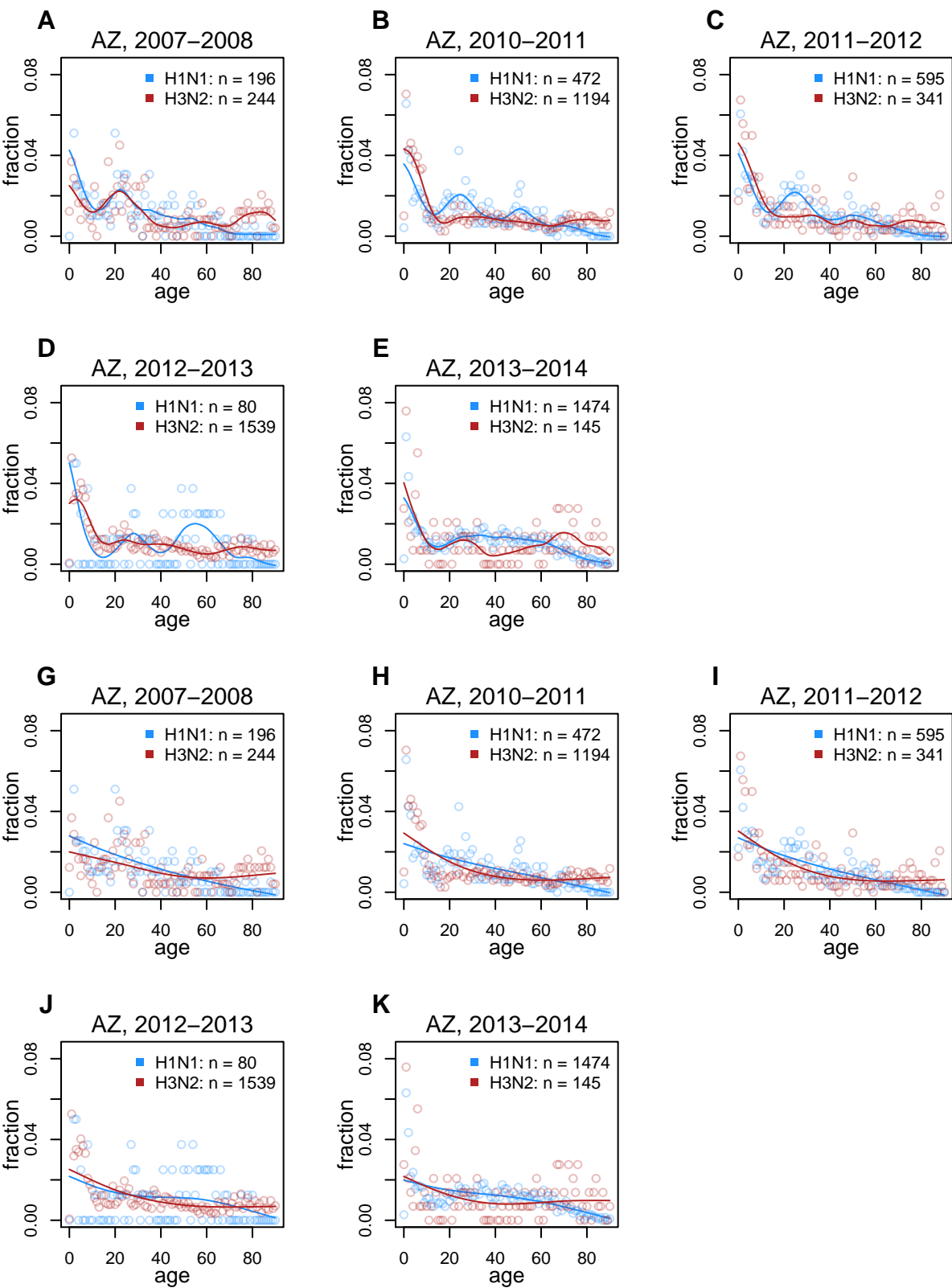

Supplement: S2 Fig — Supplement to Fig 2, with smoothing parameters chosen to fit splines that are less (A-F), or more (G-L) smooth than the splines shown in the main text. Differences between H1N1 and H3N2’s age-specific impacts remain evident, especially in the oldest cohorts, regardless of smoothness. (PDF) [file ppat.1008109.s002.pdf]

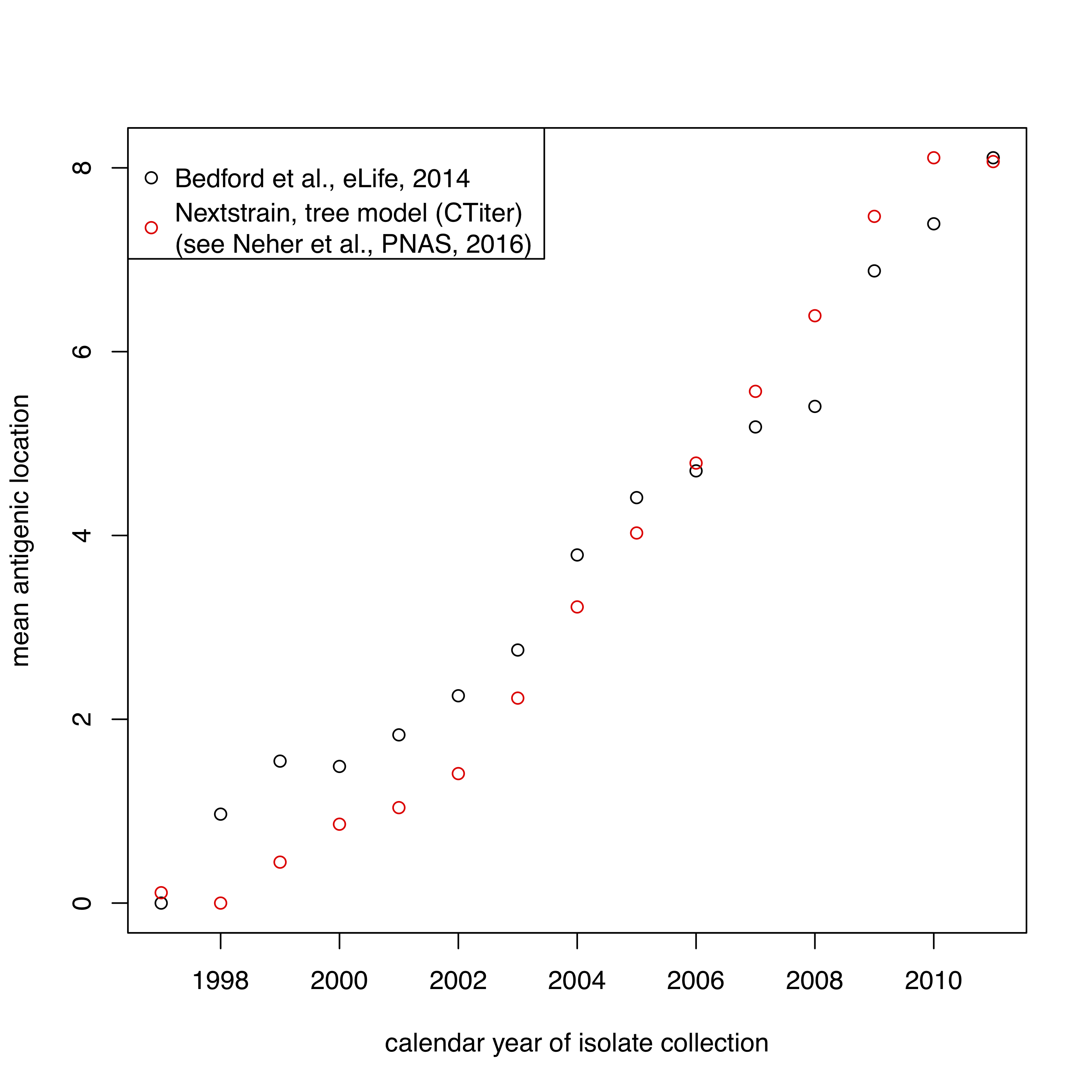

Supplement: S3 Fig — Points represent average antigenic position of all isolates from a given calendar year. (TIFF) [file ppat.1008109.s003.tiff]
